# Current status of Internet medical health service usage behavior and demand of myasthenia gravis patients: a cross-sectional study

**DOI:** 10.3389/fneur.2026.1707064

**Published:** 2026-05-21

**Authors:** Li Dong, Yaoyi Huang, Xinxian Wang, Hailing Bu, Yunying Yang, Yining Su

**Affiliations:** 1 The First Clinical Medical School of Guangzhou University of Chinese Medicine, Guangzhou, China; 2 The First Affiliated Hospital of Guangzhou University of Chinese Medicine, Guangzhou, China; 3 Guangdong Clinical Research Academy of Chinese Medicine, Guangzhou, China; 4 West China Hospital of Sichuan University, Chengdu, China

**Keywords:** chronic illness, digital health, disease management, health-related information, information-seeking behavior, Internet use, myasthenia gravis

## Abstract

**Objective:**

Internet-based medical and health services can help patients better manage chronic conditions. This study aimed to investigate the current usage, influencing factors, and needs of Internet medical health services among myasthenia gravis (MG) patients.

**Methods:**

Using a convenience sampling approach, 160 patients with myasthenia gravis who attended the First Affiliated Hospital of Guangzhou University of Chinese Medicine between February and June 2023 were recruited. Participants' baseline characteristics, current status of medical health service use, and demand for Internet medical health service and Internet disease health management services were assessed. Descriptive statistics, chi-squared tests, Fisher's exact tests, and binary logistic regression were used to examine service utilization and the factors influencing it.

**Results:**

A total of 165 questionnaires were distributed, and 160 valid questionnaires were included. Among the 160 MG patients, 105 (65.6%) were women, and 45.0% were aged ≤ 39 years. The majority of MG patients had used Internet medical health services, but their frequency of use was low. Age and privacy concerns were identified as risk factors, whereas educational level and operational convenience (ease of use) of Internet medical health service software were protective factors. MG patients reported a high demand for online appointment booking (87.5%), knowledge inquiry (78.8%), and online consultation (73.1%). The top three Internet disease management services were medication management (80.0%), symptom management (77.5%), and lifestyle management (73.1%).

**Conclusion:**

MG patients demonstrated a high willingness to use Internet-based medical and health services. However, owing to operational difficulties, privacy concerns, insufficient trust, and other barriers, MG patients have not yet fully utilized these services. Internet medical health services should prioritize highly expected functions and provide information-rich content that is easy to use.

## 1 Introduction

Myasthenia gravis (MG) is an autoimmune disorder of the neuromuscular junction characterized by fluctuating skeletal muscle weakness, prolonged disease course, and frequent recurrence (1). The reported annual incidence of MG is approximately 10–30 per million people (2, 3). Although MG is considered a rare disease, ongoing advances in diagnostic techniques have contributed to a steady increase in both its prevalence and incidence in recent years (3, 4). Despite major progress in MG research and the development of more effective therapeutic strategies since the 21st century, MG remains difficult to cure completely, and patients often require long-term disease management owing to complex symptoms, a high risk of relapse, and substantial impairments in work capacity and quality of life (5). Therefore, sustained support for symptom monitoring, medication adherence, lifestyle adjustment, and long-term follow-up is essential for the effective management of MG.

In 2023, China's National Health Commission promoted the “Internet+” healthcare model, encouraging medical institutions to leverage Internet technologies to support the long-term dynamic management of chronic diseases and reduce the reliance on hospital-based care (6). Internet-based services, which are unconstrained by time and location, have accelerated the development of healthcare and public health systems (7). Numerous studies have shown that online health information can improve treatment adherence and health outcomes by enabling patients to make more informed decisions (8–11). Patients with greater health-related knowledge also tend to demonstrate stronger disease control capacity and more effective management of treatment-related uncertainties, thereby improving overall health outcomes (12, 13). In addition, remote personalized medical support delivered via Internet-based platforms has been shown to reduce hospital readmission rates and healthcare costs, particularly among patients with recurrent symptoms and frequent hospitalization (14).

In contemporary healthcare settings, the Internet not only provides patients with health information but also offers a wide range of medical-related services (e.g., online appointment booking and medication reminders) and psychosocial support (e.g., online consultations and peer communication), which may further enhance chronic disease self-management (6, 15–17). Given the extended treatment duration and fluctuating symptom profile of MG, telehealth and Internet-based services have the potential to improve patients' ability to manage uncertainty and engage in long-term self-care. Indeed, some researchers have begun developing mobile applications for the long-term monitoring and management of MG patients (18). However, the effectiveness and sustainability of these digital health services largely depend on whether they align with patients' actual needs and expectations.

Importantly, requirement-driven frameworks have been increasingly adopted to guide the design of digital health services and self-management applications for chronic conditions. For example, a needs assessment study on spinal cord injuries systematically identified key requirements for self-management applications across various domains, such as educational information, disease and complication management, patient profiling, and technical capabilities (19). Similarly, research on inflammatory bowel disease has emphasized that the success of a self-management application depends on appropriate design and that identifying patients' essential informational needs is a critical first step in the development process (20). These studies highlight the importance of systematically assessing both functional and informational requirements from a patient perspective to support the creation of user-centered, usable, and clinically meaningful digital health platforms.

Despite growing attention to telehealth and digital health services, evidence regarding the current utilization patterns and specific needs of MG patients for Internet-based medical and health services remains limited. In particular, few studies have applied a structured requirement-analysis approach to identify the functions and disease management content that MG patients expect from such services. Therefore, this study aimed to (1) investigate the current status of Internet-based medical and health service use among MG patients, (2) identify factors associated with service utilization, and (3) explore patients' functional and chronic disease management content needs. These findings are expected to provide evidence to support the development and optimization of patient-centered Internet health services tailored to MG management.

## 2 Methods

### 2.1 Study design and participants

Our study employed a cross-sectional design and was conducted at a tertiary care, research, and teaching hospital with a muscle disease center specializing in the treatment of MG patients. MG patients who visited the hospital between February and June 2023 were recruited as participants.

The inclusion criteria were as follows: patients aged >18 years with an established diagnosis of myasthenia gravis. Participants were excluded if they (1) had other serious illnesses, such as heart failure, liver failure, kidney failure, or malignant tumors; (2) had hearing impairment or obvious visual impairment; or (3) had severe mental illness or impaired consciousness.

### 2.2 Research tools

#### 2.2.1 General information questionnaire

The general information questionnaire consists of two parts, each containing seven items. The first part assesses the patients' demographic information, including gender, age, education level, living arrangement, occupation, and payment method. The second part collects disease-related information, including MG duration, presence of comorbidities, types of daily medications (including cholinesterase inhibitors, immunosuppressants, traditional Chinese medicine for MG, and medications for other diseases), number of disease relapses in the past month, history of myasthenic crisis, thymectomy, and Myasthenia Gravis Foundation of America (MGFA) classification.

#### 2.2.2 Current status of medical health service use among MG patients

After reviewing a large body of literature related to Internet medical health services, the research team discussed and summarized the key findings and developed a questionnaire to assess the current status of Internet medical health service use among MG patients. This section of the questionnaire included two ordinal and four dichotomous items. The two ordinal items assessed patients' proficiency in smartphone use and their average daily smartphone use time (coded from 1 to 4), with higher scores indicating greater proficiency and longer daily use. The four dichotomous items assessed patients' awareness of Internet-based medical and health services, their willingness to use such services, and whether they had previously used Internet-based medical and health services.

Participants who reported prior use were further asked to complete six items regarding their current use, including usage frequency, satisfaction level, platforms used, areas of use, preferred formats for obtaining health information, and the aspects they valued the most. Participants who reported no prior use did not complete these six items; instead, they were asked to answer only one additional item regarding their main reason for not using Internet-based medical and health services.

#### 2.2.3 MG patients' Internet medical health service demand survey

Based on an investigation of commonly used Internet medical health services and a review of a large body of literature, the research team identified 12 functions of Internet medical health services: follow-up, medical health information inquiry, health report, body metrics recording, medication reminders, disease risk assessment, abnormal value alerts, health guidance, online consultation, online appointment booking, daily task check-ins, and patient communication. For each function, all items were rated on a five-point Likert scale, with five indicating “very needy” and one indicating “not needy.” When MG patients selected “5” (very needy) or “4” (quite needy), their function was considered highly desirable. The need rate for a given function was calculated as (number of “5” responses + number of “4” responses) / total number of MG patients. Cronbach's α for this section was 0.921, and the Kaiser–Meyer–Olkin measure (KMO) coefficient was 0.911, indicating good reliability and validity.

#### 2.2.4 The demand for Internet-based disease health management services among MG patients

Based on the MG-integrated Chinese–Western medicine health management program (21), we conducted a needs assessment survey covering seven primary categories (MG risk factor management, common symptom management, common complication management, common examination management, common medication management, lifestyle management, and traditional Chinese medicine (TCM) syndrome differentiation nursing management). The seven questionnaire items were rated on a five-point Likert scale, with five indicating “very needy” and one indicating “not needy.” To better understand the additional needs of MG patients, respondents were also allowed to specify the Internet-based disease management services they expected in the open-ended question section (item eight) of the survey. Cronbach's α for this section was 0.754, and the KMO coefficient was 0.778, indicating good reliability and validity.

### 2.3 Data collection

#### 2.3.1 Methodology of the survey

Data were collected from MG patients in the hospital's muscle disease outpatient and inpatient departments. Before questionnaire administration, the purpose and content of the study, as well as the principle of anonymity, were explained to the participants. Written informed consent was obtained from all participants. This study utilized a face-to-face questionnaire survey.

#### 2.3.2 Statistical methods

Data analysis was performed using the Statistical Package for Social Sciences (SPSS, IBM, NY, USA), version 26.0. Categorical variables were expressed as frequencies and percentages. Univariate analyses were conducted using chi-squared tests, with Fisher's exact test applied when the expected cell counts were <5. Multivariate analyses were performed using binary logistic regression, and statistical significance was defined as *P* < 0.05.

#### 2.3.3 Sampling

According to the Kendall sample size calculation method (22), the sample size for the demand survey is 5–10 times the number of independent variables. This questionnaire included a total of 16 independent variables, and the sample size was calculated to be approximately 80–160 cases. Accounting for a dropout rate of 10%−20%, the final sample size was 88–192 cases.

## 3 Results

### 3.1 MG patients' social demographic characteristics

A total of 165 questionnaires were distributed, and all were returned. After excluding 5 questionnaires with incomplete responses, 160 valid questionnaires were included in the final analysis. Among the 160 MG patients included in this study, women accounted for 105 (65.6%). The majority of patients were aged ≤ 39 years (45.0%). Educational attainment was mainly at the junior college level or above in 67 patients (41.9%). The majority of patients had a disease duration of ≥5 years (*n* = 85, 53.1%), and more than half of them had comorbidities (55.6%). In addition, the majority of patients were taking three or more medications for MG treatment (56.9%; Table 1).

**Table 1:** General information of myasthenia gravis patients (*N* = 160).

Variables	Group	Frequency	Percentage (%)
Gender	Men	55	34.4
	Women	105	65.6
Age	18–39	72	45.0
	40–59	67	41.9
	≥60	21	13.1
Education level	Primary and below	9	5.6
	Junior high school	43	26.9
	High school or technical secondary school	41	25.6
	Junior college and above	67	41.9
Living arrangement	Living alone	9	5.6
	Living with family	131	81.9
	Others	20	12.5
Occupation	Incumbency	74	46.3
	Out of work	53	33.1
	Retirement	33	20.6
Financial sources	Retirement or pension	31	19.4
	Family member support	49	30.6
	Wage income	66	41.3
	Others	14	8.8
Payment	Self-paying	50	31.3
	Medical insurance	91	56.9
	Others	19	11.9
Duration of MG	≤ 1 year	26	16.3
	1–5 years	49	30.6
	≥5 years	85	53.1
Comorbidity	No	89	55.6
	Yes	71	44.4
Types of daily medicines	1 type	25	15.6
	2 types	44	27.5
	≥3 types	91	56.9
Relapse over the past 1 month	No	97	60.6
	Yes	63	39.4
Experienced prior MG crisis	No	93	58.1
	Yes	67	41.9
Thymectomy	No	84	52.5
	Yes	76	47.5
MGFA classification	Type I	47	29.4
	Type II	78	48.8
	Type III	25	15.6
	Type IV	8	5.0
	Type V	2	1.3

### 3.2 Current status of Internet medical health service use by myasthenia gravis patients

The vast majority of the MG patients had heard of using the Internet to access services related to health (85.0%), three-quarters of the MG patients had used Internet medical health services (75.0%), and more than 90% of the MG patients agreed to and would be willing to use the Internet for accessing medical health services if they were adequately prepared to do so (Table 2).

**Table 2:** Current status of awareness of Internet medical health services among myasthenia gravis patients (*N* = 160).

Variables	Group	Frequency	Percentage (%)
Proficiency in smartphone operation	Not familiar	12	7.5
	Moderately familiar	53	33.1
	Quite familiar	54	33.8
	Very familiar	41	25.6
Average daily use of smartphones to access the Internet	<1 h	20	12.5
	1–2 h	30	18.8
	2–3 h	34	21.3
	**>**3 h	76	47.5
Have you heard of using the Internet to access health-related services?	Yes	136	85.0
	No	24	15.0
Willingness to use Internet medical health services	Yes	146	91.3
	No	14	8.8
Willingness to use Internet medical health services under adequate conditions	Yes	147	91.9
	No	13	8.1
Whether they have used Internet health services	Yes	120	75.0
	No	40	25.0

Among the 120 MG patients who had used Internet medical health services, more than half reported being satisfied or very satisfied (67.5%), whereas only 27 patients reported using these services frequently or always (22.5%). Only one patient reported being relatively dissatisfied. In terms of service platforms, browsers accounted for the largest proportion (34.7%) for accessing health services, while short videos and microblogs accounted for the smallest proportion (3.6%). Regarding areas of use, the online appointment booking function accounted for the largest proportion (33.8%). In terms of the format of the health information obtained, text (30.1%) and video (39.8%) were more commonly preferred. The aspects most valued by MG patients were the functions (41.8%) and operation of Internet medical health services (36.2%; Table 3).

**Table 3:** Details of the current use of Internet medical health services by MG patients.

Variables	Group	Frequency	Percentage (%)
Frequency of use of Internet medical health services	Always	10	8.3
	Frequently	17	14.2
	Sometimes	35	29.2
	Seldom	50	41.7
	Rarely	8	6.7
Use of Internet medical health services	Browser	78	34.7
	Health management software	19	8.4
	WeChat services	72	32.0
	Hospital website	48	21.3
	Short videos or microblogs	8	3.6
Areas of use of Internet medical health services	Health management knowledge	73	24.4
	Medical information	53	17.7
	Online disease counseling	54	18.1
	Online appointment booking	101	33.8
	Electronic health record	18	6.0
Satisfaction with Internet medical health services	Very satisfied	28	23.3
	Satisfied	53	44.2
	Generally satisfied	38	31.7
	Dissatisfied	1	0.8
	Very dissatisfied	0	0
Desired forms of access to health information	Text	88	30.1
	Audio	52	17.8
	Photograph	65	22.3
	Video	87	39.8
Important factors about Internet medical health services	Functions of the software	89	41.8
	Page layout of the software	36	16.9
	Operation of the software	77	36.2
	Others	11	5.2

### 3.3 Main reasons why myasthenia gravis patients have not used Internet medical health services

**Figure 1:**
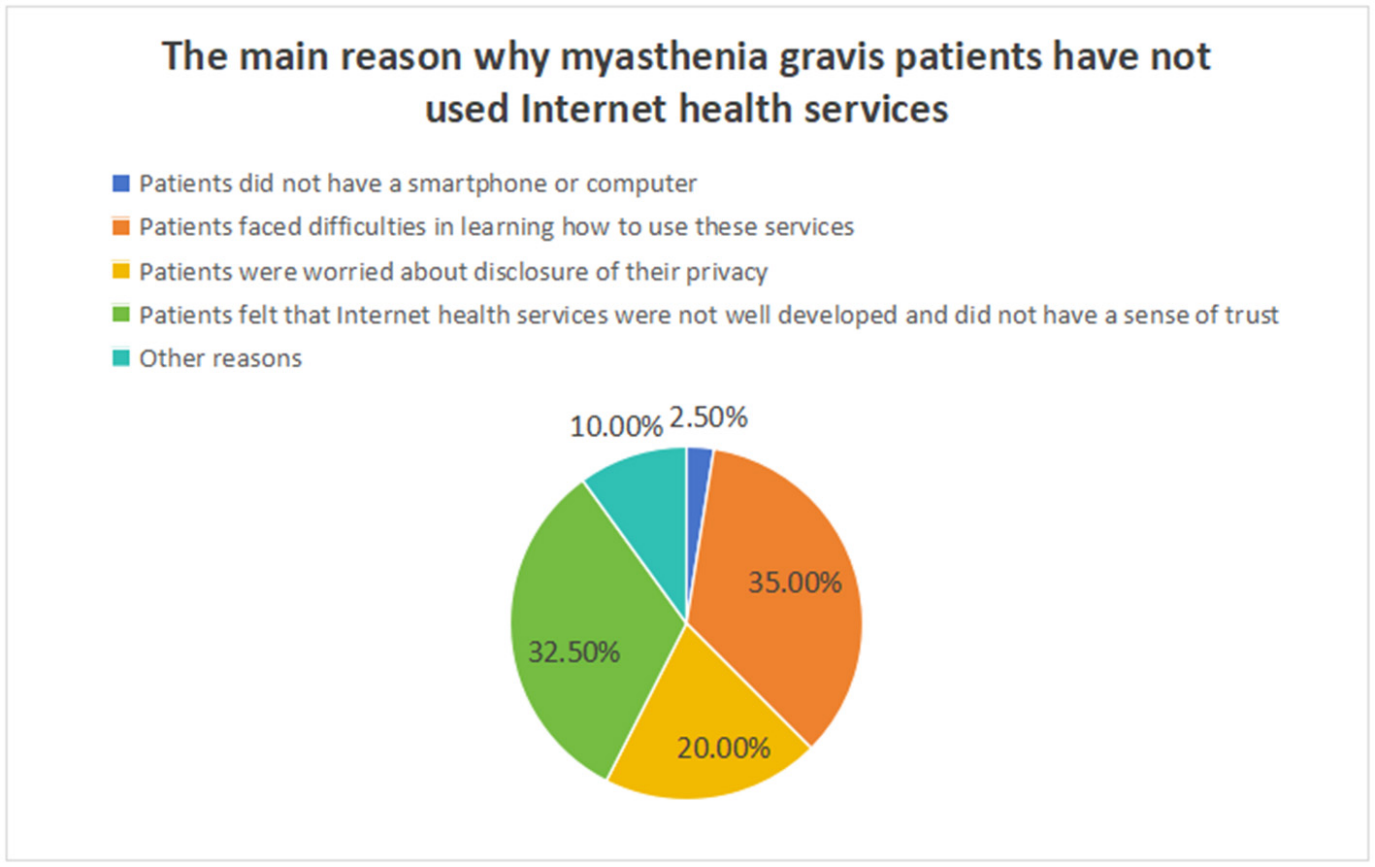
Main reasons why myasthenia gravis patients have not used Internet health services.

Regarding the main reasons why MG patients had not used Internet medical health services, the most frequently reported was that they faced difficulties in learning how to use these services (35.0%). The second reason was that they felt that the development of Internet medical health services was not yet mature and that they lacked trust in these services (32.5%). The third reason was concerns about privacy disclosure during use (20.0%). Only 2.5% of MG patients reported not using Internet medical health services because they did not have smartphones or computers (Figure 1).

### 3.4 Analysis of factors influencing the use of Internet medical health services by myasthenia gravis patients

#### 3.4.1 Univariate analysis of myasthenia gravis patients' use of Internet medical health services

The results showed that MG patients' age, education level, proficiency in operating smartphones, and average daily time spent using smartphones to access the Internet had statistically significant effects on their use of Internet medical health services (*P* < 0.001; Table 4).

**Table 4:** Univariate analysis of Internet health service use among MG patients.

Variables	Group	Whether they have used Internet health services		χ^2^	*P*-value
		No	Yes		
Gender	Men	13	42	0.083	0.773
	Women	27	78		
Age	≤ 39	4	68	27.060	<0.001^**^
	40–59	26	41		
	≥60	10	11		
Education level	Primary and below	7	2	28.177	<0.001^**^
	Junior high school	16	27		
	High school or junior college	12	29		
	3-year college and above	5	62		
Living arrangement	Living alone	2	7	2.867	0.238
	Living with family	36	95		
	Others	2	18		
Occupation	Incumbency	16	58	1.679	0.432
	Out of work	13	40		
	Retired	11	22		
Financial sources	Retirement or pension	9	22	3.531	0.317
	Family member support	16	33		
	Wage income	12	54		
	Others	3	11		
Payment	Own expense	10	40	1.444	0.486
	Medical insurance	26	65		
	Others	4	15		
Duration of MG	≤ 1 year	5	21	3.580	0.167
	1–5 years	17	32		
	≥5 years	18	67		
Comorbidity	No	19	70	1.426	0.232
	Yes	21	50		
Types of daily medicines	1	7	18	1.508	0.471
	2	8	36		
	≥3	25	66		
Relapse over the past 1 month	No	23	74	0.218	0.640
	Yes	17	46		
Experienced prior MG crisis	No	20	73	1.447	0.229
	Yes	20	47		
Thymectomy	No	21	63	0.000	1.000
	Yes	19	57		
MGFA classification	Type I	8	39	4.106^#^	0.369
	Type II	23	55		
	Type III	7	18		
	Type IV	1	7		
	Type V	1	1		
	No	Yes		
Proficiency in smartphone operation	Not familiar	8	4	26.980	<0.001^**^
	General familiarity	21	32		
	Familiar	8	46		
	Very familiar	3	38		
Average time spent using smartphones to access health services daily	<1 h	7	13	18.123	<0.001^**^
	1–2 h	15	15		
	2–3 h	9	25		
	**>**3 h	9	67		

^**^P < 0.001; ^#^Fisher's exact probability method.

#### 3.4.2 Regression analysis of the use of Internet medical health services by myasthenia gravis patients

Using whether an MG patient had used Internet medical health services as the dependent variable, the independent variables that were significant in the univariate analysis included age, education level, proficiency in smartphone operation, and the average daily time spent using smartphones to access the Internet. The Omnibus test indicated that the logistic regression model was statistically significant (*P* < 0.001). The Hosmer–Lemeshow test was not significant, suggesting a good model fit. The model correctly classified 81.9% of the cases. The logistic regression results showed that age and education level were the statistically significant factors influencing MG patients' use of Internet medical health services (*P* < 0.05; Table 5).

**Table 5:** Binary logistic regression analysis of MG patients using Internet medical health services (*N* = 160).

Variables	B	SE	χ^2^	df	*P*-value	OR	95% CI	
							Lower limit	Upper limit
Age ≤ 39 years			10.818	2	0.004^*^			
Age 40–59 years	−2.077	0.673	9.531	1	0.002^*^	0.125	0.034	0.468
Age ≥60 years	−2.471	0.844	8.566	1	0.003^*^	0.084	0.016	0.442
Junior college and above			13.254	3	0.004^*^			
Primary and below	−3.699	1.187	9.717	1	0.002^*^	0.025	0.002	0.253
Junior high school	−1.996	0.699	8.914	1	0.003^*^	0.136	0.037	0.504
High school or technical secondary school	−1.389	0.658	4.461	1	0.035^*^	0.249	0.069	0.905
Not familiar (proficiency in smartphone operation)			0.847	3	0.838			
Moderately familiar	0.223	0.815	0.075	1	0.784	1.250	0.253	6.178
Quite familiar	0.664	0.870	0.583	1	0.445	1.943	0.353	10.689
Very familiar	0.277	1.088	0.065	1	0.799	1.320	0.156	11.143
<1 h (average daily use of smartphones)			3.156	3	0.368			
1–2 h	−0.702	0.744	0.890	1	0.346	0.496	0.115	2.130
2–3 h	−0.132	0.745	0.031	1	0.860	0.877	0.203	3.778
>3 h	0.409	0.746	0.301	1	0.584	1.505	0.349	6.498
Constant	3.655	1.285	8.093	1	0.004^*^	38.664		

^*^P < 0.05; Internet health service: yes = 1, no = 0; age group: ≤ 39 years as reference; education level: undergraduate or above as reference; smartphone operation: not familiar as reference; average daily time using smartphone to access Internet: <1 h as reference.

#### 3.4.3 Survey on the requirements of myasthenia gravis patients for Internet medical health

According to a survey on functional requirements for Internet medical health services, MG patients' demand for the online appointment booking function was as high as 87.5%. The demand for health knowledge inquiry, online consultation, and health report functions exceeded 70%, whereas the demand for the task check-in function was the lowest at only 45.6% (Table 6).

**Table 6:** Functional requirements of Internet medical health for MG patients (*N* = 160).

Article	Very needy	Quite needy	Moderately needy	Slightly needy	Not needy	Percentage of demand
Follow-up function	46 (28.7)	49 (30.6)	52 (32.5)	10 (6.3)	3 (1.9)	95 (59.4)
Medical health knowledge inquiry	64 (40.0)	62 (38.8)	26 (16.3)	6 (3.8)	2 (1.3)	126 (78.8)
Health report function	61 (38.1)	54 (33.8)	35 (21.9)	7 (4.4)	3 (1.9)	115 (71.9)
Record the function of physical indicators	54 (33.8)	51 (31.9)	41 (25.6)	9 (5.6)	5 (3.1)	105 (65.6)
Disease risk assessment function	46 (28.7)	61 (38.1)	41 (25.6)	9 (5.6)	3 (1.9)	107 (66.9)
Outlier warning function	52 (32.5)	57 (35.6)	40 (25.0)	9 (5.6)	2 (1.3)	109 (68.1)
Health guidance function	49 (30.6)	58 (36.3)	44 (27.5)	7 (4.4)	2 (1.3)	107 (66.9)
Online consultation function	59 (36.9)	58 (36.3)	36 (22.5)	6 (3.8)	1 (0.6)	117 (73.1)
Online appointment booking function	96 (60.0)	44 (27.5)	16 (10.0)	3 (1.9)	1 (0.6)	140 (87.5)
Medication reminder function	46 (28.7)	46 (28.7)	34 (21.3)	19 (11.9)	15 (9.4)	92 (57.5)
Task check-ins function	29 (18.1)	44 (27.5)	52 (32.5)	24 (15.0)	11 (6.9)	73 (45.6)
Patient communication function	33 (20.6)	44 (27.5)	64 (40.0)	14 (8.8)	5 (3.1)	77 (48.1)

#### 3.4.4 Demand for Internet disease management services in MG patients

A survey on the demand for Internet disease management services in MG patients showed that the top three needs were common medication management (80.0%), common symptom management (77.5%), and lifestyle management (73.1%), followed by TCM syndrome differentiation nursing management (68.8%), common complication management (66.9%), and high-risk factor management (63.1%). The demand for common examination management was the lowest, but still exceeded 50%. In addition, MG patients hoped that these services would provide channels for selecting specialist doctors and offer live-stream educational sessions to popularize MG-related knowledge (Table 7).

**Table 7:** Demand for Internet disease management services among MG patients (*N* = 160).

Health management content	Degree of need	Order
High-risk factor management	101 (63.1)	6
Common symptom management	124 (77.5)	2
Common complications management	107 (66.9)	5
Common inspection management	87 (54.4)	7
Common medication management	128 (80.0)	1
Lifestyle management	117 (73.1)	3
TCM syndrome differentiation nursing management	110 (68.8)	4

## 4 Discussion

### 4.1 Current status of Internet-based medical and health service use among MG patients

In this study, 75.0% of patients with myasthenia gravis (MG) reported using Internet-based medical and health services, which is slightly higher than that reported in previous studies (23, 24). Nevertheless, engagement intensity remained limited, as only 22.5% of users reported using such services “always” or “often.” This pattern suggests that although digital health services have reached a relatively large proportion of MG patients, sustained or frequent engagement remains low, consistent with earlier evidence indicating that adoption does not necessarily translate into high-level use (25).

Our participants demonstrated favorable prerequisites for digital engagement: 87.5% reported using smartphones for >1 h per day, and only 7.5% were not familiar with smartphone operation. More than 90% expressed willingness to use Internet health services under adequate conditions, reflecting strong acceptance of and openness to digital health tools. However, this willingness may not fully translate into consistent use if current services fail to meet patients' expectations or remain difficult to use. Indeed, our findings showed that patients placed substantial importance on platform functionality and ease of operation, while the most common reason for non-use was difficulty learning how to use the software. This reinforces the critical role of actual user experience in shaping digital health adoption and engagement. Prior studies similarly indicate that perceived ease of use is a significant determinant of the intention to use mobile health services (26, 27).

Importantly, digital health intervention research suggests that the acceptability of and engagement with online interventions depend heavily on clarity, usability, and user-friendly delivery formats. For instance, the Internet-based Acceptance and Commitment Therapy for Inflammatory Bowel Disease (iACT4IBD) trial reported that participants valued concise and comprehensible content and emphasized the importance of accessible delivery modalities for sustained participation (28). These insights align with our observation that MG patients prioritize usability and that operational difficulties remain a major barrier to adoption.

### 4.2 Patterns of service use and preferred formats of health information

Regarding specific use domains, online appointment booking was the most frequently used function in our sample (87.5%), which is consistent with previous findings that scheduling-related services are often among the first digital healthcare functions adopted by patients (29, 30). In addition, patients reported a high demand for health knowledge inquiry (78.8%) and online consultation (73.1%), suggesting that MG patients seek both practical access-related functions and informational support through Internet platforms.

When acquiring health information, text- and video-based formats were most preferred. This preference may reflect both usability considerations and the complexity of MG-related knowledge, for which multimodal presentations may improve comprehension and facilitate self-management. Previous research has also shown that MG patients may benefit from accessing web-based health information to enhance self-management capacity and support health-related decision-making (17, 31). Collectively, these findings suggest that digital health services for MG should prioritize evidence-based content that is presented in accessible formats and integrated with key functions such as appointment booking and consultation to support long-term disease management.

### 4.3 Barriers to adoption: operational difficulty, trust, and privacy concerns

Among patients who had not used Internet medical and health services, the most common reason was difficulty learning how to use these platforms. In addition, concerns about limited service development, lack of trust, and privacy risks have been frequently reported. These barriers are highly relevant because the perceived credibility and safety of online healthcare platforms strongly influence patients' willingness to engage. Given the relatively limited regulation and quality control of online medical content, misinformation can be easily disseminated, potentially confusing or misleading MG patients (32, 33). Moreover, privacy concerns represent a major obstacle to digital health adoption, and multiple studies have shown that fear of privacy breaches reduces both the adoption and continued use of Internet medical health services (34, 35).

From an implementation perspective, addressing these barriers requires both technological solutions and patient-centered design. Research on chronic disease self-management applications highlights that privacy and security measures—such as password protection, data encryption, and transparent privacy policies—are often viewed as essential components of trustworthy digital health platforms (36). Therefore, incorporating robust privacy protection mechanisms and clearly communicating data protection strategies may be critical for enhancing MG patients' trust and promoting sustained engagement with digital health services.

### 4.4 Sociodemographic determinants of Internet health service use: age and education

Our regression analysis indicated that age and educational level were significant predictors of Internet health service use among MG patients. Older age was associated with a lower likelihood of use, which aligns with prior evidence that older adults face greater barriers to digital engagement due to reduced learning capacity, lower familiarity with technology, and difficulties adapting to new digital tools (37, 38). Although older adults who successfully adopt new technologies may experience improvements in health outcomes, self-efficacy, and wellbeing (39), they often require additional support to overcome initial barriers. These findings suggest that Internet health services should incorporate age-friendly designs, simplified interfaces, and step-by-step guidance, and they may benefit from options that support caregiver involvement to reduce barriers for older MG patients.

Similarly, lower educational attainment was associated with reduced use of Internet health services, consistent with previous research (40, 41). Patients with higher educational levels tend to be more proactive in seeking health information and using digital platforms for disease management (42). However, even high-quality online health information may contain complex terminology that can be difficult for individuals with limited health literacy to understand (43). Therefore, digital health platforms should balance professional accuracy with readability using simplified language, structured information delivery, and multimedia educational materials to enhance accessibility for MG patients with lower educational levels.

### 4.5 Functional and content needs of Internet health services among MG patients

The needs assessment in this study indicated that MG patients expressed a strong demand for functions that facilitate healthcare access and self-management, particularly online appointment booking, health knowledge inquiry, health report interpretation, and consultation. These findings reflect the long-term nature of MG care and the persistent need for ongoing medical guidance and timely support. In addition, patients reported the lowest demand for daily task check-ins, which may indicate a preference for flexible, self-directed learning and self-management rather than rigid adherence tracking.

Regarding chronic disease management, the most highly demanded services included medication management, symptom management, and lifestyle management. Given that MG patients often require multiple medications and that adherence is critical for symptom control and relapse prevention (44, 45), it is understandable that medication-related support was prioritized. Similarly, symptom monitoring and lifestyle adjustment are central components of long-term MG self-management, particularly in the context of fluctuating disease activity and uncertainty regarding symptom progression.

Notably, the demand for traditional Chinese medicine (TCM) syndrome differentiation nursing management was also relatively high (68.8%). This finding may reflect both patient interest and the recognized clinical role of TCM in MG management in China, as acknowledged in the Chinese Guidelines for the Diagnosis and Treatment of Myasthenia Gravis (2022–2024 editions) and the MG-integrated Chinese–Western medicine health management program (21, 46, 47). Therefore, this item was retained to specifically explore MG patients' needs for TCM-related nursing management. From a service design perspective, integrating evidence-based TCM-related nursing guidance into digital health platforms may help address patient needs in settings where TCM is commonly used, while ensuring that such content is presented transparently as part of a broader and more comprehensive self-management framework.

More broadly, our findings can be situated within established digital health requirement frameworks for chronic disease self-management. Studies on digital interventions for gestational diabetes have emphasized that self-management platforms should integrate educational information, medication-related support, self-management recommendations, and practical functional capabilities, enabling patients to engage in comprehensive disease management (36). Mapping MG patients' needs onto such a framework highlights the importance of developing platforms that combine strong informational components (e.g., disease knowledge, medication guidance, and symptom coping strategies) with practical service functions (e.g., appointment booking and consultation), supported by user-friendly design and privacy protection measures. Together, these strategies may enhance usability, trust, and sustained engagement among MG patients.

## 5 Limitation

This study has several limitations. First, we conducted the study in a single tertiary medical center using a convenience sampling strategy. Although the included MG patients were referred from different regions across China, the single-center design may still introduce selection bias and limit the representativeness of the sample. Second, the sample size was relatively small (*n* = 160). As myasthenia gravis is a rare disease, recruiting a large sample is inherently challenging, which may restrict the generalizability of our findings to the broader MG population. Third, due to the cross-sectional design, we cannot establish causal relationships between influencing factors and the use of Internet-based medical and health services. In addition, given the sample size constraints and the aim of capturing overall adoption among all MG patients (including frequent users, infrequent users, and non-users), we primarily examined binary usage behavior rather than engagement intensity or usage frequency. Future studies with larger multicenter samples and more diverse participant populations are warranted to validate our findings and to further apply ordinal or multinomial regression models to explore the determinants of engagement intensity and usage frequency, thereby providing more comprehensive evidence to support the development and optimization of Internet health platforms.

## 6 Conclusion

The current study found that MG patients demonstrated relatively good utilization of Internet-based medical and health services. However, due to the incomplete development of the Internet medical health service sector and issues such as inadequate privacy protection, some MG patients have not yet fully utilized these services. Therefore, Internet medical health services should strengthen privacy safeguards to enhance user trust and encourage continued engagement.

Second, the development of Internet medical health services should pay particular attention to older and less-educated MG patients, as well as those who are not proficient in operating smartphones, by providing diverse, easy-to-understand health information and user-friendly platforms with simplified operation. Furthermore, based on MG patients' Internet medical health service demand, we propose the following suggestions: while ensuring that Internet medical health services comprehensively cover essential functions and content, they should also prioritize features that MG patients value the most, such as online appointment booking, health knowledge inquiry, health report functions, and online consultation. Finally, Internet medical health services should also expand Internet disease health management services demand-related content, including medication management, common symptom management, lifestyle management, and TCM syndrome differentiation nursing management, to support MG patients in long-term disease management.

## Data Availability

The original contributions presented in the study are included in the article/supplementary material, further inquiries can be directed to the corresponding authors.
